# Monthly Digest

**Published:** 1889-02

**Authors:** 


*The Independent Practitioner.*

### MONTHLY DIGEST.

### The Trend of Dental Thought by Our Contemporaries,

FOR JANUARY.

With the opening of the New Year several changes are made in the publication of dental journals. The *Independent Practitioner* makes its first appearance under the new title of the *International*, justifying its claim to this title by an imposing array of foreign correspondents including many of the most prominent European members of the profession. The new department of Foreign Correspondents is initiated by letters from Dr. W. Bowman Macleod, Edinburg, and Dr. L. C. Bryan, Basle, Switzerland. The announcement is made that the journal will be hereafter issued on the 15th instead of the 1st of the month, thus giving a little later news than the other journals, and coming to hand when the others have lost their freshness.

A new journal makes its appearance—*The Record*, published by Hyatt, 245 East 23rd street, N. Y., for the students of the New York Dental College.

The *Dental Review* will hereafter be published by H. D. Justi.

Dr. A. H. Thompson retires from the position of associate editor of the *Western Dental Journal*, Dr. A. Morseman taking his place.

SCIENTIFIC AND THEORETICAL.

The leading article of the *International*, in the January issue, is a paper on Shock or Collapse, in Relation to Dental Operations, read by Dr. Jas. Truman before the Odontological Society of Pennsylvania, at its November meeting. The subject is exhaustively treated—physical, mental, or nervous shock, whether from anticipated or from actual suffering, from exhaustion, from the exhibition of anæsthetics, especially nitrous oxide, and in relation to both patient and operator. The effect upon the operator of the interchange of psychical influences—of constant contact with "all sorts of conditions of men, women, and children, the men all impatient, the women all nerves, the children all frightened," is graphically portrayed. The practical deduction is that protracted operations should never be allowed, unless in very exceptional cases, and that intermissions of several days should be allowed. Neither should the operator, on any consideration allow himself to be over worked to that state, too often witnessed, of complete collapse following persistent over taxing beyond the powers of endurance. The very interesting discussion of the paper is also given.

Dr. Truman advises against the extraction of a number of teeth at one sitting. Others thought that the relief of mind in knowing the operation finished will give a buoyancy to the nervous system more than balancing the shock of the operation or the dread of having to go through the ordeal again. Great importance was attached to allaying the fears of young patients, the anticipation of suffering often producing more serious results and nervous shock than the operation itself.

The *International* also publishes a paper read by Dr. J. T. Codman, before the annual meeting of the New Jersey State Society, on Climate and Diet as Affecting the Teeth, with the discussion of the same by Drs. Brown, Atkinson, Thayer, Stockton, Truman, Winder, Sudduth, Ottolengui, and others. Dr. Codman's argument, briefly stated, is that, granting that harmonious conditions will bring forth the well-developed, undiseased man, whereas we find an undeveloped and largely a physically and mentally diseased race; and knowing that man does not make his own mental and physical surroundings, *therefore* his surroundings have made him what he is, and must be inharmonious, food and climate being the great factors in his development. While admitting that the problem of our normal condition is an unsolved one, Dr. Codman believes that man was originally a fruit eater; the destruction of all vegetation during the glacial period having driven him to the abnormal practice of flesh-eating —being a creature of habit, he has remained an omnivorous feeder, but in violation of the laws of human diet. But there is a constant contest between the forces within, and their normal tendencies, and the habits forced upon him by his outward surroundings. Just as a man can be made stupid by smoke and brutal by drink, so he can be made savage and unhappy by one line of diet, or courteous, kind, and happy by another. And in correlation as our present diet and habits produce decay of the teeth, may not another diet develop and conserve them?

At the night session of the society Dr. Sudduth made a very fine lantern display of Micro-Photographs of sections of the tissues of the lower animals in various stages of embryonic life, which was followed by an interesting discussion of The Beginnings of Life, shared by Drs. Atkinson, Ottolengui, Sudduth, and others. Dr. Sudduth took the ground that the cell is the ultimate principle, the green plant being the first point in the development of life, animal life descending and blending into plant life, the *beginning* originating in creative fiat, development *de novo* being an impossibility.

Dr. Atkinson goes further back, to chaos, or the conglomerated mass of planetary substance, thence protoplasm, then protista, next the mineral kingdom of which the typical form is the crystal, next the vegetable kingdom characterized by the cell. The cell vegetates and then we come to the animal kingdom of which the primal example is not a cell, but a corpuscle—a cell and something more. The cell is simply a lump of jelly that is non-nucleated, the corpuscle differing from the cell in its promise as a body.

The *Cosmos* publishes in full, with all the tables, charts, diagrams, etc., Dr. Ottofy's valuable statistical paper on the Incipiency of Dental Caries, read before the joint meeting at Louisville, last summer.

Dr. Ottofy compares the tables compiled by Magitot, Hitchcock, Pareidt, and others, showing the variations in the results noted in different countries. He also outlines a plan by which a very thorough and systematic examination of the teeth of a thousand public school children could be made in a year, giving one hour a day to the work. Such work systematically carried out by the dentists in our large cities would prove invaluable. Dr. Ottofy's examination tables show, not only the incipiency and the prevalence of decay, but also the condition of the saliva, the density of the enamel, the presence of salivary calculus, diseases of the soft tissues, irregularities, the relative percentage of caries in the sexes and at different ages, etc.

The *Cosmos* also publishes a paper read by Dr. J. Van Dugn, Syracuse, before the Union Convention of the Dental Societies of New York, on the subject of The Dentist's Eye and Its Abnormalities. He justly styles the eye "the most important instrument which the dentist has," and emphasizes the importance of careful examination of the eyes before entering the profession of dentistry, and also of frequent periodical examinations by competent authority, disturbance often showing itself in headaches, general weariness, etc., before the eyes are suspected as the source of trouble. Dr. Van Dugn described the normal eye and the more frequent variations from normality to which it is subject, especially the different places of astigmatism. The paper was discussed by Drs. Marshall, and Brophy, of Chicago, Beers, of Montreal, and Willmott, of Toronto, Canada, and Dwinelle, of New York.

The *Cosmos* republishes from the proceedings of the Academy of Natural Sciences, a valuable illustrated paper on the Palatal Rugæ in Man, by Dr. Harrison Allen (M.D.) showing the clinical significance of the rugae in connection with catarrh, syphilis, etc., and their medico-legal value, the mucous membrane of the hard palate being almost the last tissue to be lost in the process of decomposition. The final conclusion reached is that chronic nasal catarrh is found associated with so many phases of asymmetry of the rougæ of the hard palate and the dental arches that the disease should be studied as a morbid action which is based upon morphological elements and not alone upon climatic conditions.

Dr. S. B. Palmer, in a paper read before the New York Dental Society, as reported in the *Items of Interest*, explains the Recurrence of Decay under gold or other metal fillings, and also caries under gold clasps, as a purely electro-chemical process resulting from the presence and decomposition of moisture. The organic matter of the dentine is a conductor, answering to the zinc of a battery, the filling or clasp establishes electrical polarity, contact permits an exchange of electricities, chemical action furnishing the current. The fluids in contact are electrolytes, acidity results from electrolysis, and dissolution of lime is the consequence.

In the same journal Daniel Nason, Esq., continues his valuable work on the Legal Status of Dentists. The present chapter investigates the question of legal responsibility for errors of judgment, various *causes cilebres* are cited, and the highest legal authorities quoted, showing that the standard may vary with the nature of the work required, with the condition of the patient, with locality, and other circumstances, a general rule being, "the least amount of skill with which a fair proportion of the practitioners of a given locality are endowed, is the criterion by which to judge of a professional man's ability and skill," or according to another authority, "the least amount of skill compatible with a scientific knowledge of the healing (or dental) art." A dentist may also introduce evidence with his general reputation for skillfulness, for if he shows he possesses that, the presumption will be that he used it.

DENTAL PROTECTIVE ASSOCIATION.

The journals of the month all devote more or less space to this recently chartered organization, as a rule characterizing it as a move in the right direction and possessing vast possibilities for good. The value to the profession of such a fund as it is proposed to raise, and the many ways in which it can be used, is clearly set forth by the *International.*

T. E. Weber, a Swiss dental graduate, now a senior student in the Missouri Dental College, furnishes the *Archives* some interesting points of contrast in the systems of dental education in Switzerland and the United States, as furnished by his own experience. He is especially struck with the benefits derived from the American system of quizzes, which he thinks, tends to fix in the mind "a valuable stock of clear, distinct notions," instead of allowing every thing to accumulate for the final examination, thus creating "an inextricable confusion of ideas." He also extols the prevailing spirit of "fraternity and equality which converts work into pleasure, weariness into perseverance, shirking into energy."

In the same journal Dr. W. H. Johnston, Brooklyn, deplores that lack of *appreciation* displayed by that large class of dentists who attend dental society meetings, accepting the good the gods provide *free* but who never join a society, or in any other way contribute to the common good. It is discouraging to those who, year after year, stand in the breach and sacrifice liberally both time and money—and yet, as the puplic judges rather by the lack of knowledge in the mass rather than by the high attainments of the few, the latter, for the honor of the profession, must labor for the enlightment of the former, or suffer in the general estimate of the whole.

HISTORICAL.

The *Items of Interest* puplishes an interesting letter from Dr. A. W. Baxley giving a history of the efforts by which, through the personal solicitations of Dr. Chapin A. Harris and himself the charter of the Baltimore College was obtained from the legislature of the State of Maryland, and of the opening of that institution in the winter of 1840, with a Faculty of four professors—all graduates in medicine—and a class of five legitimate students. In view of the fine buildings now occupied by this venerable institution after a lapse of nearly a half century it is interesting to learn that the first series of lectures were delivered in a single small room, while from prudential considerations, the teaching of practical anatomy was relegated to a secluded stable loft!

THE BORN DENTIST.

In the same journal Dr. R. L. Cochran says that education will never make a man a dentist unless he is born a dentist. He must be born with a base on which to build with natural faculties to be cultivated, powers that can be developed, education only serving to make mind and hand work together.

Dr. C. I. Peterson takes issue with the statement that dentists are born, not made, and says that those who have attained to the highest eminence are those whose minds are a storehouse of knowledge, rich pure thought, obtained only after long years of patient study and discipline.

Dr. C. S. Butler, as reported in the *Cosmos*, read before the Union Convention of the Fifth and Eighth District Societies of New York, a paper on the Pathological Conditions of the Mouth Due to Artificial Dentures. The presence of such a foreign body in the oral cavity is always liable to become an irritant to the tissues with which it comes in contact. The exfoliated epithelial cells and the excreta of the mucous glands, owing to the higher temperature, if a non-conducting material is used, are shed too rapidly, and aphthæ result, or if close fitting, are not flushed away by the saliva, and decomposed. If the air is exhausted too great pressure may cause serious results, mechanical irritation from poor adaptation is also a fruitful source of abnormal results; also the fungi developed through lack of cleanliness.

Dr. C. C. Carroll thought much of the trouble observed was due to electrical action. Dr. Brockway attributes much to the substances collecting on the surface of the plates; Dr. Barrett to the fermentation of such substances which become a medium for the pure culture of bacteria, aphthea being simply colonie of microbes. Dr. S. B. Palmer thinks the electric action pointed out by Dr. Carrol, converts the substances mentioned by Dr. Brockway into the acids which generate the bacteria mentioned by Dr. Barrett.

The chapter of Dr. Talbot's work on Irregularities given in the January issue of the *Cosmos* treats of The Dental Arch, placing it on a different base from the usual definition. He considers each jaw as consisting of two separate arches, of which the central incisor is the anterior base, and the first molar the posterior base, the cuspid being the corner-stone, coming into place last either locking the arch, holding all the teeth in proper position, or, if space is lacking, with its long roots and great leverage, forcing the other teeth into the different malpositions we are called upon to regulate. The second and third molars he relegates to the position of abutments to either arch. He also combats the stereotyped ideas in regard to the relation between V-shaped and saddle-shaped arches and idiocy, and thumb-sucking as a factor in irregularities, considering these as due entirely to inharmonious development of teeth and jaws, the maxillary bones having gradually diminished in size with the lapse of ages the teeth remaining the same, normally that they were three thousand years ago.

Dr. J. H. Woolley, Chicago, in the *Archives*, discusses the questions of Reflex Action and Reflex Neurosis in connection with dental irritation, especially with pulpless teeth, citing numerous interesting cases in support of his argument. Recognizing in pulpless teeth, improperly treated, or not treated at all, the source of countless aberrations from healthy functional activity in other parts of the body. Dr. Woolley emphasizes the importance of their proper treatment and filling, with a view to permanency in the jaw, placing them in healthy relation to all contiguous tissues.

Dr. G. A. Bowman, in the same journal, deals with the question of Conservatism in Dental Practice, which, he says, touches the circle at every point where there is an enemy to the highest state of perfection. Among the threatening foes he enumerates erosion, caries, receding gums, degeneration of alveolar process, want of occlusion, imperfectly adjusted artificial attachments, etc. In the treatment of the first two lesions he gives the post of honor to Dr. W. Storer How's "inlays" as an artistic development in repairs in labial surfaces, and to copper amalgam for saving qualities in cavities in buccal surfaces. $H^2O^2$ and sulphur A take the lead in vanquishing the next two lesions named, with the suggestion of campho-phenique as a germicide. The bridge is indicated as pre-eminently suited to remedy the fifth condition.

The *International* publishes (from advance sheets of the 12th edition of Harris' Principles and Practice) portions of a valuable illustrated article by Dr. W. G. A. Bonwill on Regulators and Methods of correcting Irregularities. Among the principles laid down he advises, 1st—To commence as soon as possible after the seventh year, or after evidence of decided irregularity, and, 2nd—To watch all children's teeth from the third year and determine by an exploring needle every three months the exact position of the coming permanent teeth as soon as the first permanent molar has appeared.

In the treatment of irregularities Dr. Bonwill advocates fixed apparatus that the patient can not interfere with, and continuous pressure, studying the case always from plaster impressions and models in the anatomical articulation by which means all movements may be studied. The temporary molars are utilized as fulcrums as no injury is inflicted by cutting and grooving teeth which will soon be lost, ligatures are kept from impinging upon the gums by gutta-percha or thin oxy-phosphate. The normal standard of lap of superior incisors and cuspids or underbite, is governed by the length of the cusps of the first superior bicuspid. The illustrations given show, first, a series of applications of the spiral spring as used by Dr. Bonwill previous to 1863, and recently reproduced as the "Talbot Spiral Spring;" also a series of far more simple appliances now used by Dr. Bonwill.

OPERATIVE DENTISTRY—PRACTICAL POINTS.

In the New York Odontological Society Dr. Bonwill defined the true philosophy of Contour Operations, the main points being to wedge well apart, and to extend the periphery of the filling far beyond the boundary line where tooth structures might possibly approximate.

Dr. E. S. Gaylord, New Haven, in the *Archives*, offers a valuable suggestion in regard to the retention of Cement Fillings. Having noted what has doubtless struck every one handling the cements, how tenaciously the leavings adhere to the mixing slab on which the fluid is dropped before the powder is added, it occurred to him to paint the entire cavity with the fluid before inserting the plastic mass. The filling adheres so thoroughly as to require a burr or drill for subsequent removal. In cementing metal bands or crowns, etching the surface of the metal and painting with the fluid gives the same desirable result.

Dr. W. E. Driscoll, Manatee, Fla., in *Items of Interest*, states that he leaves a few fibres of cotton in the creamy cement with which he fills the apex of root canals, packing in this way till a point is reached where cement alone can be used to advantage. This has been his successful practice for fifteen years.

Dr. E. C. Bryan, Pittsfield, Maine, in *Archives*, advocates Combination Fillings, as a saving of labor to the dentist, of suffering and exhaustion to the patient, and of expense to both, besides preserving the teeth better. He fills the cavity from one to two-thirds full with tin, Slayton's felt or Robinson's fibrous foil, facing with gold.

Dr. J. W. Canaday, Albany, New York, in the same journal, advocates the combination of tin and gold, in immature teeth of poor structure, especially those of nervous, impatient children. He folds a sheet of No. 4 tin foil within a sheet of No. 4 cohesive gold, completely enclosing it, folding to the thickness of No. 48 gold, cutting in ribbons which are slightly warmed and folded within the cavity, condensed first by hand and then with a steel burnisher in the engine. After a few months, fillings of this combination become as hard as the best amalgam, with clean margins and no crevicing, while the teeth will be found greatly improved in texture.

Dr. Osmun, in the discussion of this paper before the Central Dental Association of Northern New Jersey, said that we have but a very faint conception of the possibilities in store for us when we shall understand more about the combination of metals in filling. Amalgam and gold, tin and gold, felt foil and gold, all give better results than gold alone, though the reason has not yet been made clear.

Dr. Thayer uses very light tin—2 grains to the sheet—rolled in small pellets and covered with five or six thicknesses of No. 4 gold. This combination gives a permanency of corners and margins not obtained by other method.

Dr. Rhein is skeptical as to the extreme value placed on the combination, having seen many failures in work coming from the best operators. He doubts the therapeutic effects in immature teeth.

Dr. Osmun thinks the good results observed are due to the closer mechanical joint made, which is also true of copper amalgam.

Dr. Rhein draws the line distinctly between the improvement in the character of young teeth, due to functional activity, and that observed later in life, when we can not look for therapeutic effects from filling materials, such as the combinations under discussion.

Dr. Luckey cited a case in which great apparent benefit resulted from the insertion of a pin head pellet of amalgam in the centre of a large gold filling in a compound crown cavity extending down the distal surface, a little hole having been burred in the gold for the insertion of the amalgam at the time of making the filling. The amalgam was as black as ink, but the gold was bright as a new dollar. In all the other teeth in the same mouth, not treated in this manner, the margins had been patched, but in this one the margins were perfect.

Dr. Canaday finds that where only a small portion of tin is used, it disintegrates forming a fine black powder.

### CLEANLINESS.

Dr. Wm. N. Morrison, in the Iowa State Society (*Items of Interest*) advises that temporary stoppings be put in the teeth of new patients to test the care likely to be taken of our work, educating them before putting in our best work. If hands, nails, ears, etc., are not clean how can we expect them to keep their mouths clean--our work will be thrown away. Plenty of pure water and a good toothbrush are the only essentials for the elimination of acids and germs.

Dr. J. B. Rogers insists upon the use of the toothpick and thinks that one reason why caries is more prevalent in the teeth of ladies is because men use the toothpick while reading the paper, or on the way to business, while a lady sits down to some occupation which employs her hands. Patients should be taught how to clean the teeth.

CARELESSNESS.

Dr. H. H. Harrison, Cadiz, Ohio, in *Archives*, says that failures due to want of knowledge are inexcusable; if due to want of time, that should have been overcome; if to lack of compensation, that should not have been considered; if to want of conscience, they are malicious and unpardonable. Each operation should be such that we would be willing to submit to an expert as the record of our professional ability.

COCAINE.

Dr. Henley, Texas, relates in the *Items of Interest*, some of his earlier unfortunate experiences with cocaine, due to fermentation of the solution, to excess of carbolic acid as a preservative, etc. He now uses, with invariable satisfaction, Parke, Davis & Co's Crystals in 5 grain vials, dividing the contents roughly among four small vials, to one of which he adds about ten drops of water when wanted for a case in hand, making a fresh solution for each case.

Dr. A. H. Hilzim, Jackson, Miss., in the *Archives*, criticizes severely Dr. J. C. Reese's Instructions for the Use of Cocaine, (published in November *Archives*) and thinks that if generally followed patients would be landed in the country where teeth are not needed. He quotes from various authorities as to the toxic effects recorded, the alarming symptoms, the antidotes, etc. He uses cocaine daily in his practice, but with the precautions observed in administering a powerful drug. He prepares it as follows, using the German preparation of C. F. Boehringer & Son, Manheim: Add two drops deliquesced carbolic acid to 100 drops distilled water, in which dissolve 4 grains cocaine muriate. He first paints the gum, and then injects into the gum holding the cheek and lips well away.

SULPHUR IN DENTISTRY.

Dr. Horace Dean, Jersey City, First District Society New York, in the *Cosmos*, gave the result of his experiments in the use of sulphur in the treatment of root canals and as a cement.

He fuses the sulphur in the platinum loop of the electric cautery, introducing it into the root canal of putrescent pulps while still fuming. He finds that it materially shortens the time necessary to restore inflamed and abscessed roots to health with an almost immediate cessation of pain. He also finds the fumes to be a valuable obtundent for sensitive dentine. Used as a lining for shallow cavities, or with thin walls of enamel, it serves to make the gold adhere to tooth substance where undercuts are inadmissible.

In crown operations it forms a valuable cement for the pin; the canal should be dried and made as hot as is safe and pleasant; it is then smeared with melted sulphur by means of the platinum loop. The pin is sulphur-cemented into the crown, and the hot pin coated with melted sulphur and pressed home. It can at any time be removed by heating the beaks of a small pair of forceps thus softening the cement. Many other uses for this disinfectant cement are suggested.

Dr. L. C. Bryan, Basle, Switzerland, in the *International*, gives the following method of preparing an opaque black wax for articulating crowns: one part, by bulk, of lampblack and five parts white or yellow wax are melted together and rolled into sticks. The crown and root being ground to fit approximately, a small piece of this wax warmed, is placed around the pin and the crown placed in position. The points requiring grinding will be very accurately indicated, the amount to be removed being estimated by "sounding" the wax with a fine point.

Dr. Bonwill, in the New York Otontological Society, (*Items of Interest* from *Cosmos*) uttered a word of timely warning against the too common sacrifice of natural tooth-crowns for artificial substitutes. Contouring with good amalgam, capping pulps when necessary with oxyphosphate, will save a large percentage of badly diseased teeth in less time, and at less expense and inconvenience to the patient than caps or crowns of gold. Very large compound fillings in teeth with dead pulps should be anchored with pins in the roots.

In the Union Convention of the Sixth, Seventh, and Eighth District Dental Society, New York, as reported in the *Cosmos*, Dr. J. H. Beebe described an ingenious method of casting the grinding surface of a crown to the articulation, in the case of a crown adapted to an inferior molar which was abraded on the coronal surface by an unusually large cusp on the upper tooth, the patient objecting to having the cusp ground away.

Dr. E. Parmley Brown, in the First District Dental Society, stated that he found trouble in getting sufficient gas-pressure in Europe to fuse American body, but English teeth and bodies bake beautifully in the Verrier furnace; it is objectional, however, on account of the density of the coloring matter, which gives an undesirable opacity to the finished work.

Dr. Brown has perfected a cement which is like glass around the tooth, withstands the action of the oral secretions, and can be put in and taken out with heat.

PULP CAPPING.

Dr. J. A. Robinson, Jackson, Mich., in *Archives*, reports almost invariable success in capping aching, exposed pulps in all stages of inflammation in deciduous teeth with oxyphosphate mixed with carbolic acid, first bathing the pulp with chloroform. He makes no separation between approximal cavities in adjacent teeth of very young children. Teeth thus treated have been refilled in more than one case, and the pulp been found alive, in one case even bleeding after three years under the cap. As he says, such practice is far in advance of destroying pulps with arsenic, and elevates the profession, being more in harmony with conscientious practice. He is emphatic as to the importance of taking off all occlusion from such teeth.

IMPLANTATION.

Dr. Ottolengui, in the *Cosmos*, gives the rules which govern him in the selection of teeth for implantation, and to which, together with the use of proper instruments, he attributes his success, having had no failures thus far, with a record of thirty cases. He submits all teeth to close examination with a lens, which often reveals incipient exostosis or absorption in apparently perfect teeth. He rejects all such and all having calcified pulps, and abraded or eroded crown, or softening at the neck from recession of gums, and as a rule uses teeth only within a week after extraction.

Illustrations of three noteworthy cases show the great valve of implantation.

In the *Archives* Dr. Ottolengui describes his treatment o a severe case of pyorrhea alveolaris. The disease had attacked the mouth at the four ends of the dental arches. On the right side above it had been checked by the timely removal of the wisdom tooth, and below by the extraction of the sixth-year molars, the second molars being diseased. At the upper left corner the wisdom tooth was badly diseased, and also the adjacent second molar on the distal side. The wisdom tooth was so loose that it could be moved readily, and even pushed up into its socket. To bind it firmly a deep wide groove was cut in the crowns of the three molars in which a piece of gold plate was laid, gold filling being first placed across the floor of the groove, and also built around and over the bar, with gold and platinum.

The second molars below, which stood alone and were very loose, were held firmly in place by a band fitting around the molar and bicusid, having spurs burnished into groves cut in the crowns, and the intervening space filled with a molar backed with gold plate, the three pieces soldered together and further secured by gold screws passing through the bands entering the substance of the tooth. This might be called the Van Woert-Rhei-Ottolengui method, being the result of a combination of suggestions. The patient was thus enabled to eat on these teeth for the first time in four years. There were no deposits; the medical treatment consisted of Robinson's remedy, hydrogen peroxide and bichloride of mercury, followed by dressings of tannin and glycerine. Hydro-napthol, 1 to 300, was also used.

PROSTHETIC DENTISTRY.

With one notable exception prosthetic dentistry is given, as usual, but small space in the journals of the month.

The *Ohio Journal*, however, publishes in full a very valuable paper, read before the Odontological Society of Indianapolis, by Dr. L. N. Comstock, with photographic reproductions of his drawings and sketches, including a manikn with movable cuts illustrating the results of neglecting to observe or correct a great variety of variations from the normal standard of proportions. The paper is entitled The Artistic Adaptation of Artificial Dentures, and shows that beyond the possession of mechanical skill the prosthetic dentist must also be an artist, an anatomist, a physiognomist. He must have cultivated perceptive faculties for form, locality, size, proportion, movement, grace, beauty, color; a knowledge of artistic anatomy, classic outline, and the philosophy of expression. He should also be draughtsman enough to draw all the different points noted in a careful study of the face from all points and under varying expressions, and his outfit should include pencils, brushes, palette and colors.

In the same journal Dr. Haskell shows that the combination of continuous gum and rubber recently recommended is a revival of a method tried and condemned as worthless a quarter of a century since.

At the November meeting of the First District Society, New York, *Cosmos*, Dr. Rhein presented the patient wearing a piece of all-porcelain bridgework extending from the superior left first molar to the lateral and central incisor roots, with the exception of a sound cuspid which was covered with an enameled platinum crown. This piece is described on page 245 of Dr. Evans' Treatise on Crown and Bridgework.

Mr. E. E. Clark demonstrated the various uses to which the Ward electro-metallic dental plates can be put in the shape of bridges, crowns, and plates.

Dr. F. C. Green, New Albany, suggests in the *Archives*, in cases of extreme absorption of the inferior alveolus, taking the final impression in a cup made from a first plaster impression, trimmed out on the edges and where it impinges on the muscles, wetting this plaster "cup" very thoroughly before using it as such. If carefully done a plate will result that will not rise or rattle while speaking.

### NEW APPLIANCES.

Very few new appliances are mentioned in the January journals. Dr. Ottolengui's instruments for implantation—tubular knife for incising the gum tissues, reamers for the sockets, etc.—are figured and described in the *Cosmos*. Dr. W. G. Deane has a new tooth-crown mandrel for cutting and facing all forms of porcelain crowns, the mandrel holding stones for cutting concave, corners or flat and hollowed recess for the protection of the pin while grinding the crown.

Dr. J. W. Ivory has a large variety of improved clamps; also Dr. Elliot's separator and matrix holder.

S. S. White & Co. has a new, large and heavy driving wheel for the engine by which the ordinary speed can be attained by a much slower movement of the treadle, making the stop quicker and easier.

Taggart's Corundum Point and Disk Maker is coming into very general use giving universal satisfaction. Harwood's New Nitrous Oxide Blowpipe is now on the market, at the very low price of $10.

Though not exactly an appliance, Hirsh's Operating Coats will be appreciated by every dentist who wants a well-made, well-fitting operating coat of good material—bleached drilling—at $7 the half-dozen.

An elegant little novelty is the Ideal Holder, or drip tray, for the prophylactic tooth brushes of the Florence Co. One should be found on every washstand.

